# Supplementary material for: Using mobile EEG to study auditory work strain during simulated surgical procedures
Source: Sci Rep. 2024 Oct 14;14:24026. doi: 10.1038/s41598-024-74946-9 (PMC11473642; doi:10.1038/s41598-024-74946-9)
Supplement: Supplementary file 1 — Supplementary Information. [file 41598_2024_74946_MOESM1_ESM.pdf]

Supplementary Material: Using mobile EEG to study auditory work strain during simulated surgical procedures.

Marc Rosenkranz, Thorge Haupt, Manuela Jaeger, Verena N.Uslar, Martin G. Bleichner

Deviations from preregistration

TRF analyses

- Initially we ran the cross-validation procedure 100 times and randomly chose 90% of the blocks as test data and the remaining 10% of the blocks as training data. However, we realized that this is computationally expensive while resulting in very similar prediction values as with the approach in the manuscript.
- During the last step of TRF calculation we computed the window from -220 to 500 ms. In the preregistration the window ranged from -200 to 500 ms, however, due to an early artefact common to TRF calculations[52] we had to increase the window.
- Due to an oversight we did not detail in the preregistration how we determined the individual optimal lambda value.
- We z-standardized the TRF values before GED analyses. This was necessary as the individual lambda range was large, thus resulting in individual TRFs on different scales which lead to violations of the normality assumption for the statistical comparison.

GED analyses Our initial idea was to individualize the analyzes pipeline, especially the GED computation and compute a spatial filter per participant. However, it turned out that this worked well for our pilot data but to a much lesser degree for the entire dataset. The GED analyses led to component time-series and maps that were hard to interpret or not meaningful making component selection difficult. Therefore, we used a grand average GED model and made the following adaptations:

- We used the GED weights calculated from all subjects and applied them to each subject and block. The initial idea was to calculate GED weights for each participant calculated from the grand average over blocks and apply the weights to all blocks of of this participant.
- We calculated fixed time-windows to determine the component time-windows. Initially, the full width at half maximum/minimum with respect to the peak was used, but this lead to time-windows that extended over the response of interest.
- We calculated the S and R covariance matrix for each participant and cleaned the matrix before averaging. This is a step included in Cohen (2022)[55]. Initially, we used the grand average ERP of a participant to compute one S and one R matrix, thereby making the cleaning step unnecessary.

Further adaptions to the GED analyses were made:

- Initially the peak of all ERP and TRF components were searched for in the same time-window. However, it turned out that the envelope response peaks earlier than the ERPs and TRFs calculated from the onsets due to temporal smearing. To account for this, we searched for the envelope peaks in an earlier time-window, as stated in the manuscript.
- In the preregistration the lambda value to regularize the R matrix was defined as $\lambda = 0.1$ which was a typo. The initial analyses was set up with $\lambda = 0.01$ as stated in the manuscript and which was also used in Cohen (2022)[55].

Statistical analyse

- The initial idea was to calculate the maximum LMM (i.e., $\hat{y} \sim condition + (condition|participant)$). However, in most cases this model did not converge, especially when adding time as a predictor. We decided to start with model 1, then successively add fixed effects, and finally evaluate the change in model fit.
- For the memory scores, we initially wanted to calculate a beta regression model, but realized that these are not applicable when responses are bound to 0 or 1, which was the case for our data.

Shift of letter onset

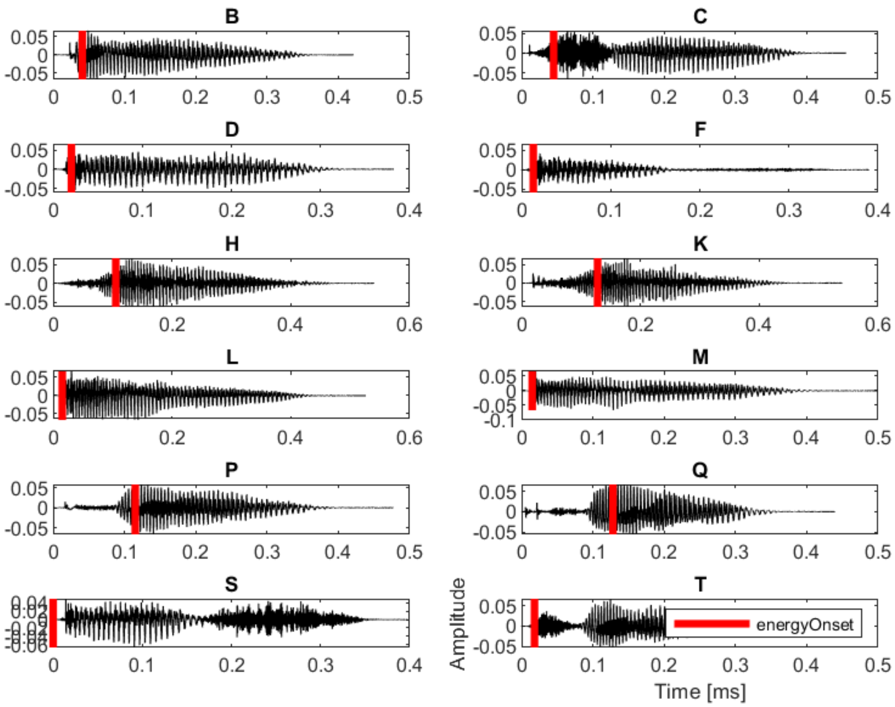

Figure S 1. Time-representation of each letter. The red line indiactes the shift of the letter onset.

Removed IC containing muscle movement

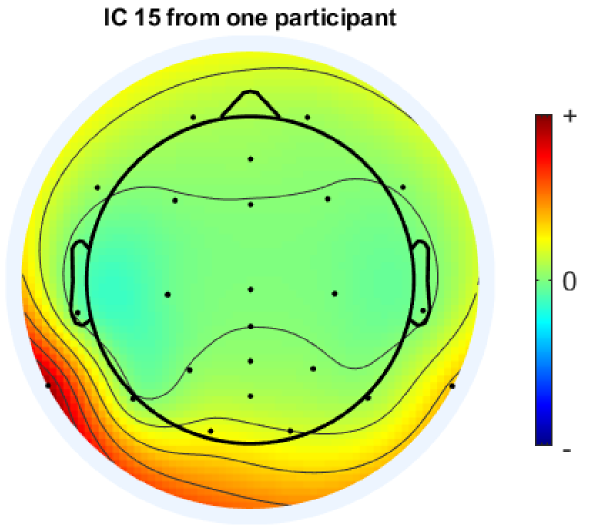

Figure S 2. This is an example of an independent component of one participant that was manually removed, as it contained noise at the mastoid electrode.

GED covariance matrices

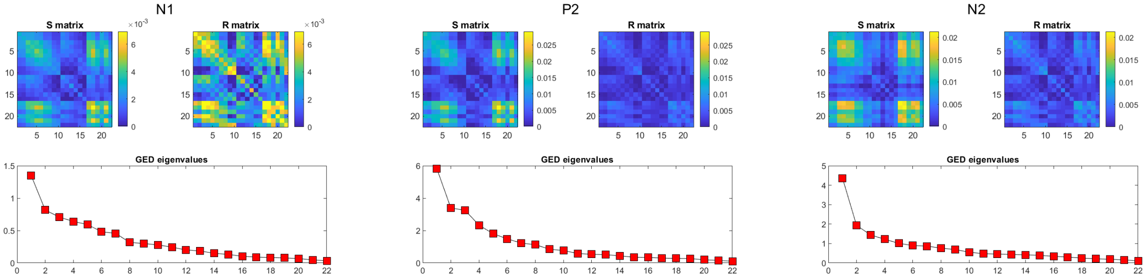

(a) ERP components in response to the letters.

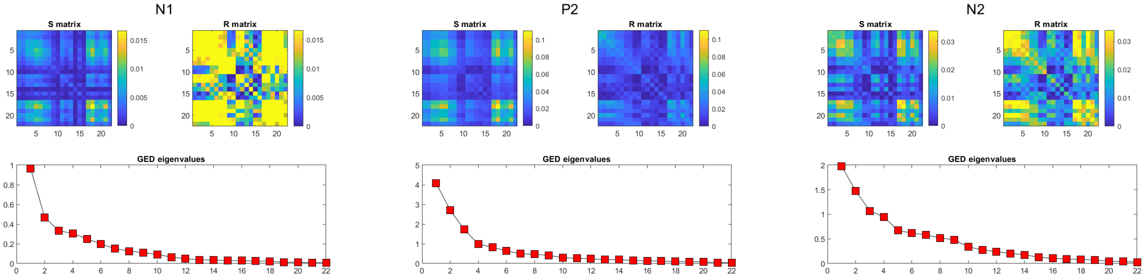

(b) TRF components calculated from the noise reduced envelope.

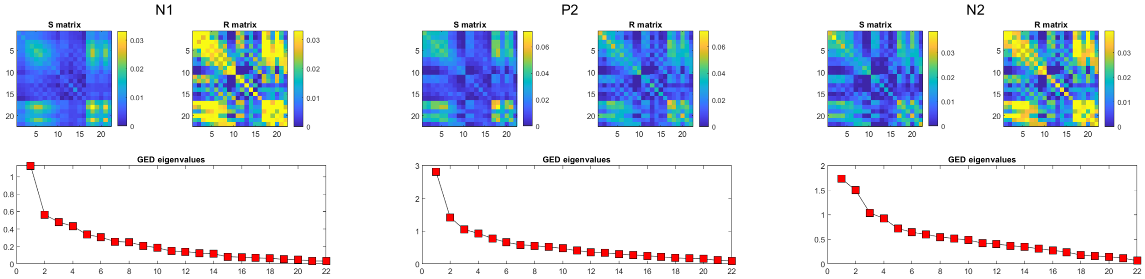

(c) TRF components calculated from the noise reduced envelope.

Figure S 3. GED covariance matrices and eigenvalues for (A) ERPs, and TRFs calculated from the (B) noise reduced envelope and (C) onsets. Upper left and right plot depict the $\bar{S}$ and $\bar{R}$ covariance matrix, respectively. The lower plot depicts the eigenvalues of each component.

GED components

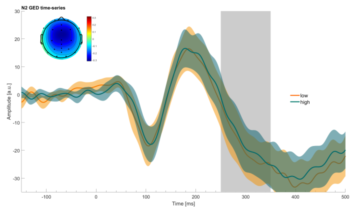

(a) ERP N2: First GED component.

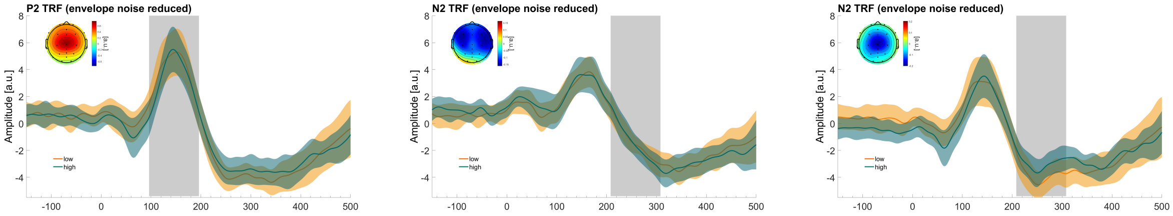

(b) **(left)** First GED component of P2 TRF envelope (noise-reduced). **(middle)** First GED component of N2 TRF envelope (noise-reduced) and **(right)** second GED component of N2 TRF envelope (noise-reduced). The second component was chose for the N2 analyses, as the map and topographies look more plausible.

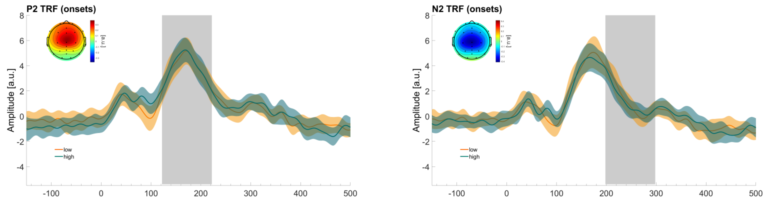

(c) **(left)** First GED component of P2 TRF onset. **(middle)** First GED component of N2 TRF onset.

Figure S 4. This figure shows GED components that were analyzed but not shown in the manuscript. The GED time-course displays averaged data (line) and standard error (shaded area) across participants. The grey area shows the time-window that was used to compute the average amplitude. The topographies depict the forward model of the GED component.

Statistical model compairsons

Behavioral and subjective Results

response	predictor	AIC	BIC	logLik	Chisq	Df	Pr(>Chisq)
tlx-effort	condition	3164.4	3182.1	-1578.2			
	condition+time	3166.0	3188.2	-1578.0	0.3593	1	0.5489
	condition*time	3166.7	3193.3	-1577.3	1.3156	1	0.2514
tlx-effort	condition	3440.7	3458.5	-1716.4			
	condition+time	3442.5	3464.7	-1716.2	0.2661	1	0.6059
	condition*time	3444.4	3471.0	-1716.2	0.0961	1	0.7565
tlx-distraction	condition	3006.0	3023.7	-1499.0			
	condition+time	3006.4	3028.7	-1498.2	1.5193	1	0.2177
	condition*time	3007.8	3034.5	-1497.9	0.6005	1	0.4384
Performance time	condition	5656.2	5674.0	-2824.1			
	condition+time	5576.4	5598.6	-2783.2	81.8695	1	<2e-16 ***
	condition*time	5577.7	5604.3	-2782.8	0.6821	1	0.4089
Mistakes	condition	619.97	633.29	-306.98			
	condition+time	613.99	631.74	-302.99	7.9818	1	0.004725 **
	condition*time	614.66	636.86	-302.33	1.3249	1	0.249721
Tissue damage	condition	3807.9	3821.2	-1901.0			
	condition+time	3778.8	3796.6	-1885.4	31.0797	1	2.476e-08 ***
	condition*time	3778.8	3801.0	-1884.4	1.9995	1	0.1573

Table S 2. The table shows model performance of the statistical models. The simplest model contained condition as a predictor. There is no model comparison for the memory condition, as the models did not converge when adding time as a predictor. Signif. codes: 0 '***' 0.001 '**' 0.01 '*' 0.05 '.' 0.1 ' ' 1

EEG data

response	predictor	AIC	BIC	logLik	Chisq	Df	Pr(>Chisq)
ERP - N1	condition	5484.0	5501.5	-2738.0			
	condition+time	5475.5	5497.4	-2732.7	10.4946	1	0.001197 **
	condition*time	5476.2	5502.6	-2732.1	1.2575	1	0.262118
ERP - P2	condition	5549.9 5567.5	-2770.9	5541.9			
	condition+time	5541.7	5563.7	-2765.9	10.1876	1	0.001414 **
	condition*time	5543.3	5569.6	-2765.6	0.4539	1	0.500484
ERP - N2	condition	5622.4	5639.9	-2807.2			
	condition+time	5623.6	5645.6	-2806.8	0.7320	1	0.3922
	condition*time	5625.5	5651.9	-2806.8	0.0959	1	0.7568
TRFenv - N1	condition	4067.9	4085.5	-2030			
	condition+time	4066.6	4088.6	-2028.3	3.2459	1	0.0716 .
	condition*time	4068.2	4094.6	-2028.1	0.4148	1	0.5196
TRFenv - P2	condition	3887.3	3904.9	-1939.4			
	condition+time	3889.2	3911.2	-1939.6	0.0451	1	0.8318
	condition*time	3891.2	3917.6	-1938.6	0.0131	1	0.9090
TRFenv - N2	condition	3778.3	3795.9	-1885.2			
	condition+time	3780.3	3802.3	-1885.1	0.0175	1	0.8946
	condition*time	3782.3	3808.6	-1885.1	0.0258	1	0.8723
TRFons - N1	condition	3663.6	3681.2	-1827.8			
	condition+time	3659.2	3681.2	-1824.6	6.3816	1	0.01153 *
	condition*time	3661.2	3681.5	-1824.6	0.0408	1	0.83994
TRFons - P2	condition	3610.4	3628.0	-1801.2			
	condition+time	3610.1	3632.1	-1800.1	2.2199	1	0.1362
	condition*time	3610.6	3637.0	1799.3	1.5038	1	0.2201
TRFons - N2	condition	3440.1	3457.7	-1716.0			
	condition+time	3439.8	3461.8	-1714.9	2.2371	1	0.1347
	condition*time	3441.8	3468.2	-1714.9	0.0232	1	0.8790

Table S 3. The table shows model performance of the statistical models. The simplest model contained condition as a predictor. TRFenv: TRFs were calculated using the noise reduced envelope. TRFons: TRFs were calculated using the onsets of the raw audio. Signif. codes: 0 ‘***’ 0.001 ‘**’ 0.01 ‘*’ 0.05 ‘.’ 0.1 ‘ ’ 1
